# Elevated expression of the RNA‐binding motif protein 43 predicts poor prognosis in esophageal squamous cell carcinoma

**DOI:** 10.1007/s10147-021-01976-y

**Published:** 2021-08-16

**Authors:** Yong Li, Li-Li Liu, Rui Hu, Qi Sun, Xiao-Bo Wen, Rong-Zhen Luo, Shu-Mei Yan

**Affiliations:** 1 Sun Yat-Sen University Cancer Center, State Key Laboratory of Oncology in South China, Collaborative Innovation Center for Cancer Medicine, Guangzhou, 510060 China; 2 Department of Pathology, Sun Yat-Sen University Cancer Center, 651# Dong Feng Road East, Guangzhou, 510060 Guangdong China; 3 The First Affiliated Hospital, College of Medicine, Zhejiang University, Hangzhou, 310022 China

**Keywords:** RBM43, Esophageal squamous cell carcinoma, Immunohistochemistry, Prognosis

## Abstract

RNA-binding proteins (RBPs) play crucial roles in the post-transcriptional regulation of mRNA during numerous physiological and pathological processes, including tumor genesis and development. However, the role of RNA-binding motif protein 43 (RBM43) in esophageal squamous cell carcinoma (ESCC) has not been reported so far. The current study was the first to evaluate RBM43 protein expression by immunohistochemistry (IHC) in an independent cohort of 207 patients with ESCC, to explore its potential prognostic value and clinical relevance in ESCC. The results indicated that RBM43 protein levels were significantly elevated in ESCC tissues and increased RBM43 expression was associated with age and N categories. In addition, ESCC patients with high expression of RBM43 had shorter overall survival (OS) and disease‐free survival (DFS) than those with low RBM43 expression. Furthermore, when survival analyses were conducted at different clinical stages, overexpression of RBM43 was significantly correlated with shortened survival in patients with ESCC at early stages (TNM stage I–II and N0 stage). Cox regression analysis further proved that high RBM43 expression was an independent predictor of poor prognosis in ESCC patients. In conclusion, increased expression of RBM43 is correlated with malignant attributes to ESCC and predicts unfavorable prognosis, suggesting an effective prognostic biomarker and potential therapeutic target for ESCC.

## Introduction

According to the GLOBOCAN 2018 database, the esophageal cancer (EC) is a common malignant tumor ranked the seventh and sixth for incidence and mortality rate, respectively, in the world [1]. Esophageal cancer is further subdivided into two principal subtypes: esophageal adenocarcinoma (EA) and esophageal squamous cell carcinoma (ESCC). Compared with EA, ESCC is more common in China, accounting for nearly half of the newly diagnosed cases and deaths worldwide, and > 90% of patients are diagnosed at a middle or late stage [2–4]. Great progress of the treatments, such as surgical resection, radiotherapy, chemotherapy, and immunotherapy, has been made for ESCC, whereas clinical outcome of locally advanced ESCC remains disappointed with a 5-year survival rate not exceeding 30% in China [5]. Therefore, it is critical to explore effective biomarkers and develop more accurate prognostic models for ESCC patients.

RNA-binding proteins (RBPs) are crucial post-transcriptional regulators of gene expression with key roles in numerous cellular pathways and biological processes^[5,6]^. Since they contain ≥ 1 RNA‑binding domains (RBDs) or motifs (RBMs), RBPs can recognize target RNAs and regulate all aspects of RNA metabolism and function, including RNA splicing, translation, localization and stability [7–9]. Given these functions, it is not surprising that the abnormal changes in RBPs are associated with cancer development and progression [10–13]. For example, RBM38 and RBM24, as relatively extensively investigated RBPs, frequently function tumor-suppressive role in various human cancer types through forming negative feedback loop with tumor suppressors (TS) such as p53 family (i.e., p53, p63, p73) [14–17]. In addition, RNA-binding protein CELF1 is overexpressed in colorectal cancer and promotes cell migration, invasion, and chemoresistance by targeting ETS2 [18].

RNA-binding motif protein 43 (RBM43), as a member of the RNA-binding motif protein (RBM) family, contains two RNA recognition motifs (RRMs), RNP1 and RNP2. Some previous studies have revealed that RBM43 acts as a genetic factor that contributes to type 2 diabetes (T2DM) [19], endometriosis, esophageal adenocarcinoma (EA), and Barrett’s esophagus (BE) [20]. However, the expression of RBM43 and its clinical significance in ESCC have not been reported to the best of our knowledge. In this manuscript, we explored the association between the expression levels of RBM43 with clinicopathological characteristics and overall prognosis for the first time, using immunohistochemistry (IHC) in 207 pairs of ESCC tissues and adjacent non-tumor tissues from Sun Yat-sen University Cancer Center (SYSUCC).

## Materials and methods

### Patients and tissue specimens

This study was approved by the medical ethics committee of Sun Yat-sen University Cancer Center. A total of 207 ESCC tissues and adjacent non-tumor tissues were sectioned and confirmed by pathologic review for IHC. Patient selection criteria were as follows: (a) confirmed primary ESCC by pathology; (b) underwent surgery without any prior treatment; (c) with no other previous or synchronous malignancy; (d) frozen tissues, complete clinical information and long-term follow-up data were available. The survival status of all 207 patients was reconfirmed in May 2012.

### Immunohistochemistry

The tumor and adjacent normal tissues from ESCC patients were fixed with 10% paraformaldehyde, then embedded in paraffin. The paraffin sections were incubated at 55 °C for 4 h, then deparaffinized in xylene and rehydrated in a graded ethanol series. Treated sections were washed three times with phosphate-buffered saline (PBS) and then boiled in antigen retrieval buffer for 15 min using a microwave oven to perform the heating antigen retrieval. Sections were treated with 3% hydrogen peroxide to quench endogenous peroxidase activity, followed by block nonspecific binding using 1% bovine serum albumin. Then the sections were incubated with rabbit anti-human polyclonal antibody against RBM43 (1:100 dilution; PA5-24,106; thermofisher) at 4 °C overnight in a moist chamber. Blocking solution alone was applied as a negative control. Finally, the slides were incubated with horseradish peroxidase (HRP) for 30 min at 37 °C, followed by addition of chromogen 3,3′-diaminobenzidine (DAB) for visualization.

### Evaluation of IHC

Two independent pathologists (S.-M.Y, L.-L.L) blinded to clinicopathological data assessed the immunoreactivity scores (IRS) for all stained sections. A final IRS (range: 0–300) for each case was obtained by multiplying the intensity score (0 = none; 1 = weak; 2 = moderate; 3 = strong) and the percentage of positive tumor cells (range: 0–100%). All these ESCC patients were divided into low and high expression groups according to the median value of IRS system.

### Statistical analysis

The receiver operating characteristic (ROC) method was applied to define the RBM43 IRS cutoff value. All statistical analyses were performed using SPSS software (SPSS, version 18.0). The correlations between RBM43 expression and clinicopathologic features were analyzed using Pearson’s *χ*^2^ test. Overall survival (OS) and disease-free survival (DFS) was assessed using the Kaplan–Meier method and compared by the log-rank test. Univariate and multivariate analyses to identify independent prognostic factors were performed using the Cox regression model.

## Results

### Patient characteristics

Table 1 shows the clinicopathological parameters of 207 patients with ESCC, including 151 males and 56 females, aged 32 from 80 years (median 57 years).

**Table 1:** RBM43 expression and clinicopathologic variables of 207 esophageal squamous cell carcinoma cases

Variable	RBM43 expression	Low	High	*p* Value^a^
	Cases (*n* = 207)	(*n* = 99, percent)	(*n* = 108, percent)	
Age^b^ (years)				0.003
≤ 57	104	39 (18.8)	65 (31.4)	
> 57	103	60 (29.0)	43 (20.8)	
Sex				0.703
Female	56	28 (13.5)	28 (13.5)	
Male	151	71 (34.3)	80 (38.6)	
Tumor location				0.571
Upper	10	4 (1.9)	6 (2.9)	
Middle	139	70 (33.8)	69 (33.3)	
Lower	58	25 (12.1)	33 (15.9)	
Histological grade^c^				0.812
Grade 1	49	24 (11.6)	25 (12.1)	
Grade 2	134	65 (31.4)	69 (33.3)	
Grade 3	24	10 (4.8)	14 (6.8)	
P T status^c^				0.13
p T 1	6	5 (2.4)	1 (0.5)	
p T 2	48	18 (8.7)	30 (14.5)	
p T 3	150	74 (35.7)	76 (36.7)	
p T 4	3	2 (1.0)	1 (0.5)	
N categories				0.038
Negative	112	61 (29.5)	51 (24.6)	
Positive	95	38 (18.4)	57 (27.5)	
TNM stage^c^				0.247
Stage I	8	5 (2.4)	3 (1.4)	
Stage II	119	61 (29.5)	58 (28.0)	
Stage III	80	33 (15.9)	47 (22.7)	

^a^Probability value of < 0.05 indicates statistical significance. Probability values are calculated by Pearson’s c2 test

^b^Age is divided according to the median age of 57 years

^c^The grading and histopathology stage of ESCC specimens are based on the World Health Organization (WHO) classification published in 2009

### Immunostaining for RBM43

**Fig. 1:**
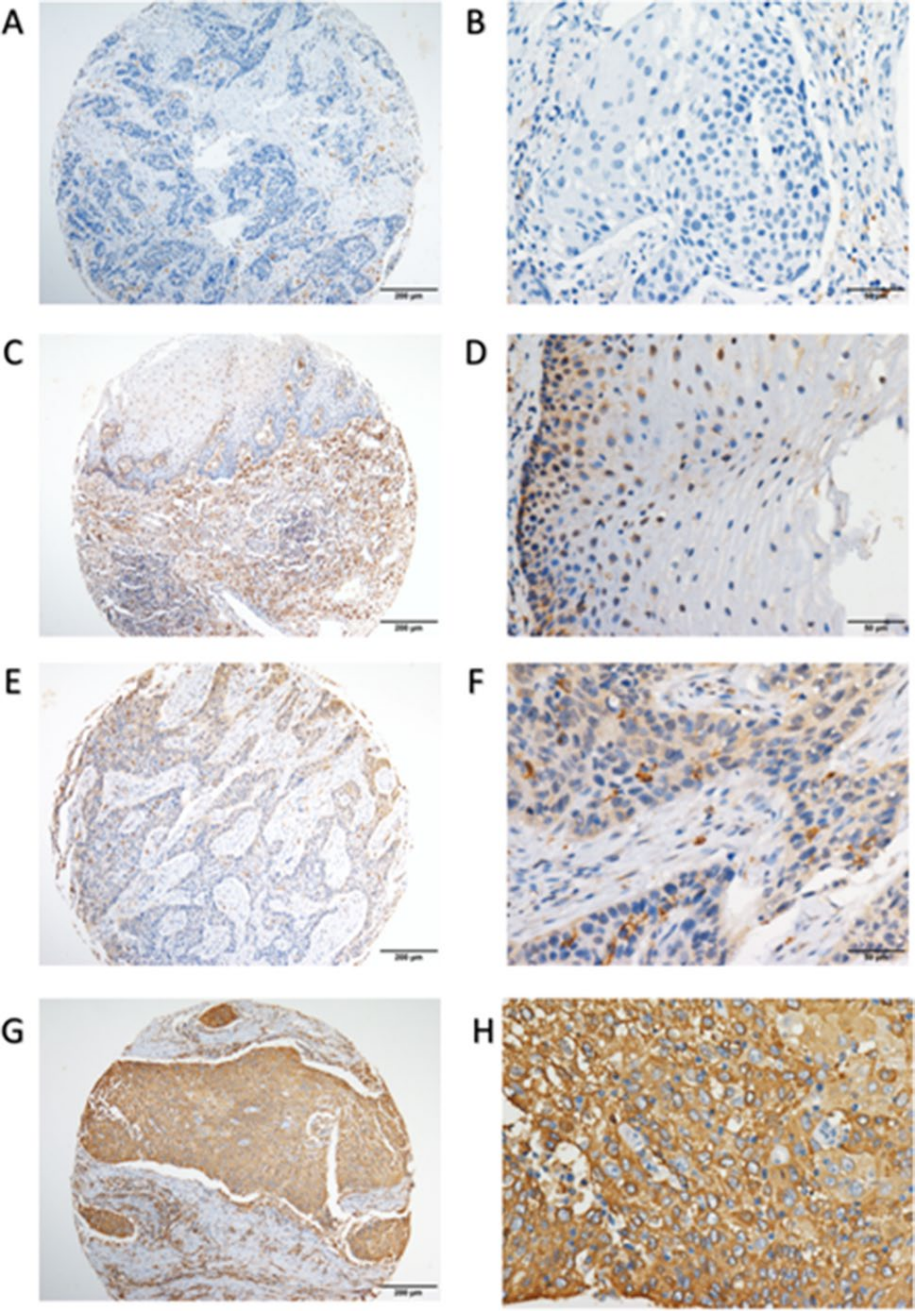
RBM43 protein expression in ESCC tissues and adjacent normal tissues, as detected by immunohistochemistry staining. (**a**, **b**) Representative image of negative RBM43 protein expression in adjacent normal tissues (magnification: **a**, × 100; **b**, × 400). (**c**, **d**) Representative image of low RBM43 expression in adjacent normal tissues (magnification: **c**, × 100; **d**, × 400). (**e**, **f**) Representative image of low RBM43 expression in ESCC tissues (magnification: **e**, × 100; **f**, × 400). (**g**, **h**) Representative image of high RBM43 expression in ESCC tissues (magnification: **g**, × 100; **h**, × 400)

**Fig. 2:**
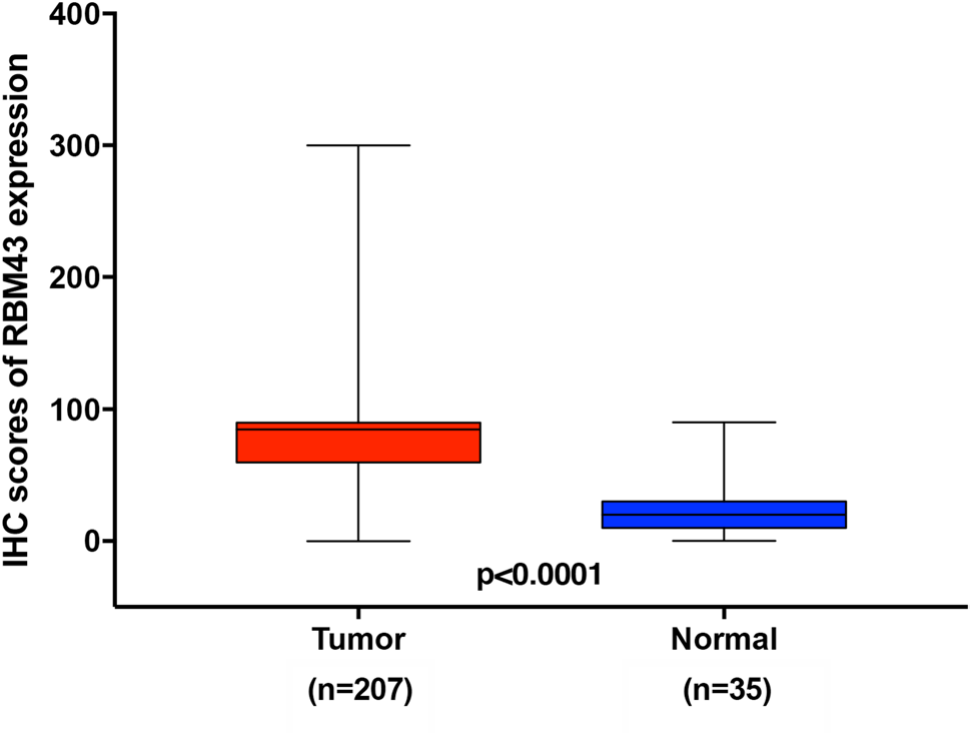
Levels of RBM43 expression were analysed in ESCC tissues (n = 207) and adjacent normal tissues (*n* = 35) using IHC

The IRS cutoff value for RBM43 expression was determined by the ROC curve. For the present study, the IRS cutoff value was 85. Thus, expression greater than this value was considered high and, otherwise, low. The results of IHC staining revealed that the expression of RBM43 protein was predominantly localized to cytoplasm of ESCC tissues, whereas no or weak staining was observed in adjacent non-tumor tissues (Fig. 1). In addition, we have found that RBM43 protein can also be expressed in stromal cells within the tumor microenvironment (TME). However, in light of the abundance and complexity of RBM43 expression patterns within ESCC cells and the corresponding stroma, we have chosen to focus the discussion on expression of RBM43 in tumor cells. The analysis of IRS showed that RBM43 was commonly upregulated in ESCC issues, compared with adjacent non-tumor tissues (*p* < 0.0001, Fig. 2).

### RBM43 expression and clinicopathological features

To further characterize the roles of RBM43 in ESCC, the relationships between RBM43 expression and clinicopathologic parameters are summarized in Table 1. The RBM43 expression was significantly associated with age (*p* = 0.030) and N categories (*p* = 0.038). No statistically significant association existed between RBM43 expression and sex, tumor location, histological grade, pT status, TNM Stage (*p* = 0.703, 0.571, 0.812, 0.130, 0.249, respectively, Table 1).

### RBM43 expression and survival

Among the 207 ESCC patients, the median follow-up time was 41 months (range, 4–115 months), and none were lost to follow-up. By the end of follow‐up, 114 (55.1%) patients died of ESCC, while 93 (44.9%) patients survived. The 5-year OS and DFS for the entire cohort were 49.9% and 46.4%, with median times of 58 and 48 months, respectively.

**Fig. 3:**
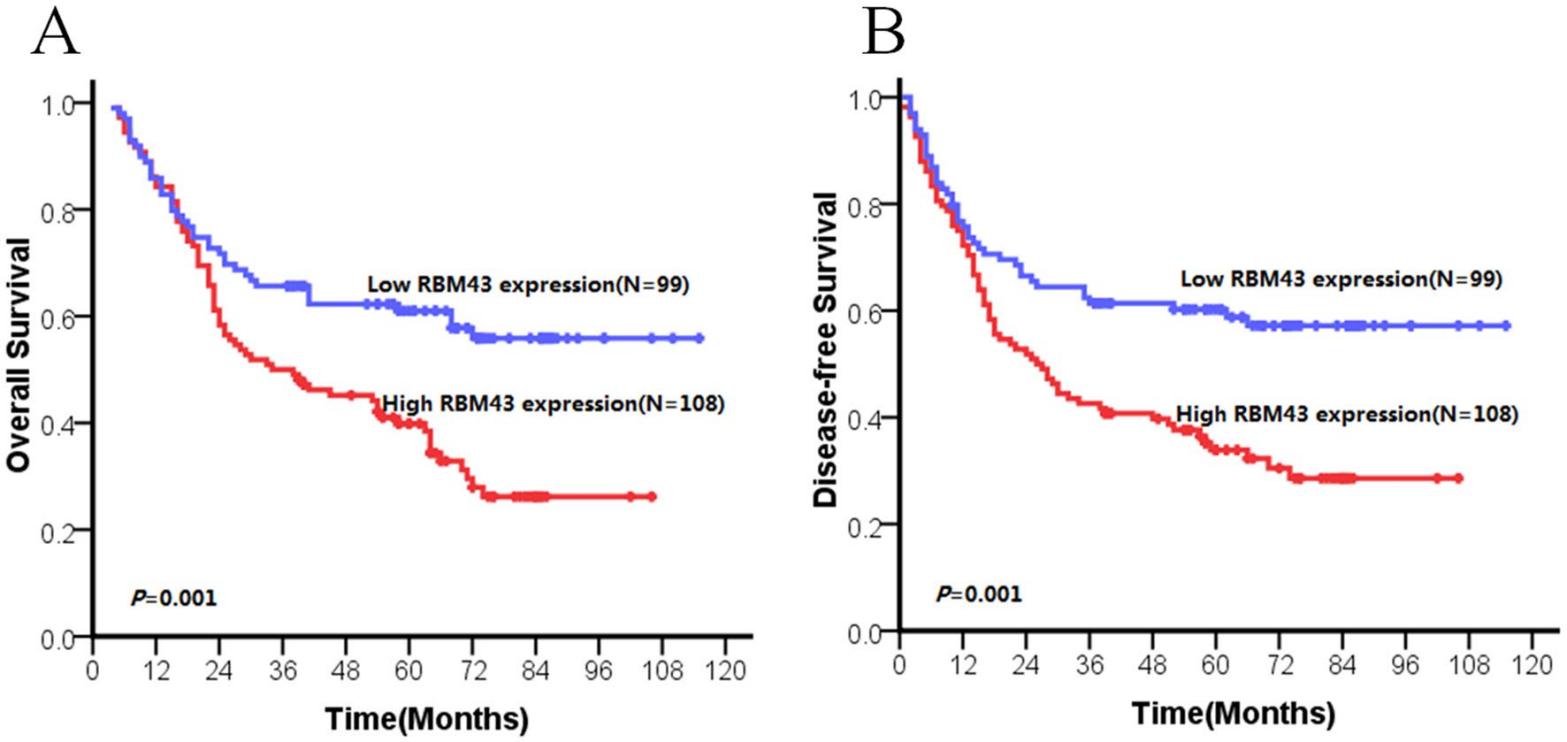
OS and DFS curves of patients with ESCC based on their RBM43 expression. (**a**) OS curves: all patients with low and high RBM43 expression levels. (**b**) DFS curves: all patients with low and high RBM43 expression levels

**Fig. 4:**
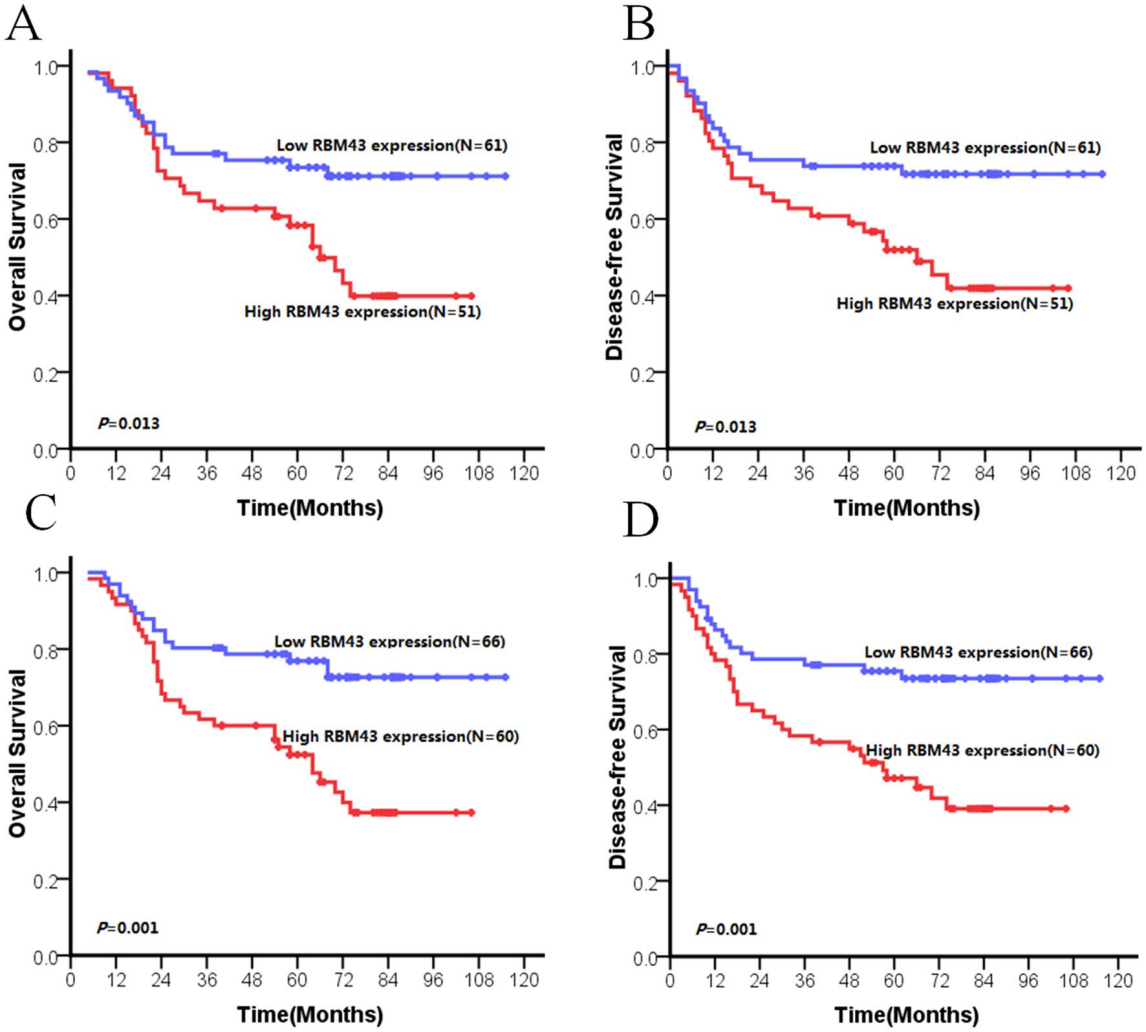
Subgroup analysis of patients with ESCC based on their RBM43 expression. (**a**) OS curves: patients in TNM stage I–II with high and low levels of RBM43 expression. (**b**) DFS curves: patients in TNM stage I–II with high and low levels of RBM43 expression. (**c**) OS curves: patients in N0 stage with high and low levels of RBM43 expression. (**d**) DFS curves: patients in N0 stage with high and low levels of RBM43 expression

The present data revealed that patients with high RBM43 expression had significantly shorter survival than those with low RBM43 expression (*p* = 0.001 for OS and DFS, Fig. 3, Table 2). Furthermore, the prognostic value of RBM43 expression in different subgroups of ESCC patients was examined according to the 8th edition of the TNM (tumor, node, metastasis) classification. We found a significant correlation between high RBM43 expression levels and decreased survival in TNM stage I–II (*p* = 0.013 for OS and DFS, Fig. 4a, b, Table 2) and lymph node metastasis-negative subgroup (*p* = 0.01 for OS and DFS, Fig. 4c, d, Table 2).

**Table 2:** RBM43 expression in ESCC patients by Kaplan–Meier survival analysis (log-rank test)

Variable	Case	DFS (months)			OS (months)		
		Mean	Median	*p* value	Mean	Median	*p* value
Total				0.001			0.001
Low expression	99	73.1	NR		75.2	NR	
High expression	108	46.2	26		50.7	34	
pT categories							
pT1-2				0.008			0.006
Low expression	23	86.1	NR		88.8	NR	
High expression	31	51	51		55.8	55	
pT3-4				0.013			0.016
Low expression	76	67.9	NR		69.8	NR	
High expression	77	44.3	22		48.5	28	
pN categories							
pN = 0				0.013			0.013
Low expression	61	87	NR		89	NR	
High expression	51	62	66		65.3	66	
pN = 1/2/3				0.145			0.193
Low expression	38	41.6	25		44.5	30	
High expression	57	28.5	16		34.4	24	
Histologic grade							
G1				0.01			0.009
Low expression	24	81.8	NR		83.3	NR	
High expression	25	47.6	39		52.1	54	
G2-3				0.013			0.016
Low expression	75	69.2	NR		71.4	NR	
High expression	83	45.8	24		50.3	30	
pTNM categories							
I + II				0.001			0.001
Low expression	66	89.6	NR		91.7	NR	
High expression	60	58.5	57		62.3	64	
III				0.521			0.832
Low expression	33	32.2	13		34.8	19	
High expression	47	25	15		31.4	23	

*ESCC* esophageal squamous cell carcinoma; *DFS* disease free survival; *OS* overall survival; *NR* not reached

### Univariate and multivariate Cox regression analyses

The predictive roles of RBM43 in ESCC prognosis were further assessed by Cox regression analysis. The univariate analysis showed that the following parameters were significantly associated with OS: RBM43 expression, tumor status, nodal status (*p* = 0.001, *p* = 0.016 and *p* < 0.001, respectively, Table 3). Furthermore, a multivariate Cox regression analysis was performed for these factors. The results demonstrated that RBM43 expression, tumor status and nodal status were independent prognostic factors for ESCC (*p* = 0.012, *p* = 0.029 and *p* < 0.001, respectively, Table 3). Similarly, the analysis in DFS data also confirmed the role of RBM43 expression as an independent prognostic predictor for ESCC (*p* = 0.006; Table 4). Thus, high expression of RBM43 may predict poor prognosis in patients with ESCC.

**Table 3:** Univariate and multivariate analyses of overall survival of ESCC patients

	Univariate analyses			Multivariate analyses		
	HR	(95%CI)	*p* value	HR	(95%CI)	*p* value
Age (years), (> 57 vs. ≤ 57)	1.052	0.729–1.520	0.785			
Gender (male vs. female)	1.262	0.828–1.925	0.280			
Tumor location (upper/middle/lower)	0.931	0.650–1.334	0.697			
Histological grade, (G3/G2/G1)	1.356	0.986–1.865	0.061			
Tumor status (T4/T3/T2/T1)	1.640	1.096–2.453	0.016*	1.575	1.047–2.370	0.029*
Nodal status (N > 0/N0)	2.980	2.030–4.376	< 0.001*	2.717	1.839–4.012	< 0.001*
RBM43 expression, (High/Low)	1.896	1.291–2.784	0.001*	1.641	1.113–2.420	0.012*

*ESCC*: esophageal squamous cell carcinoma; *p < 0.05, statistically significant

**Table 4:** Univariate and multivariate analyses of disease-free survival of ESCC patients

Variables						
	HR	(95%CI)	*p* value	HR	(95%CI)	*p* value
Age (years), (> 57 vs. ≤ 57)	1.050	0.727–1.517	0.793			
Gender (male vs. female)	1.203	0.789–1.835	0.390			
Tumor location (upper/middle/lower)	0.900	0.630–1.285	0.561			
Histological grade, (G3/G2/G1)	1.389	1.009–1.912	0.044*	1.340	0.972–1.847	0.074
Tumor status (T4/T3/T2/T1)	1.545	1.033–2.309	0.034*	1.508	0.999–2.274	0.05*
Nodal status (N > 0/N0)	2.859	1.947–4.198	< 0.001*	2.569	1.742–3.789	< 0.001*
RBM43 expression, (High/Low)	1.901	1.295–2.791	0.001*	1.713	1.163–2.522	0.006*

*ESCC* esophageal squamous cell carcinoma; *p < 0.05, statistically significant

## Discussion

Recently, an increasing number of studies have reported that RNA-binding proteins (RBPs) are widely dysregulated in numerous human cancers and play an important part in tumor development and progression [21, 22]. A number of current RBPs studies in cancer were conducted using IHC staining, trying to explore the associations between the changes of RBPs expression and patient prognosis. For instance, it revealed that the expression of RBM47 was significantly elevated in non-small-cell lung cancer (NSCLC) tissues and the overexpression of RBM47 promoted NSCLC progression and metastasis, indicating that RBM47 may be a potential biomarker and therapeutic target for NSCLC [23]. In addition, in invasive breast cancer, high RBM3 expression was found to act as a favorable prognostic indicator with prolonged survival and showed a significant correlation with less aggressive phenotypes [24]. Apart from aberrant protein expression levels, attention has also been directed toward the wide range of biological functions of RBPs ranging from RNA processing and splicing, translation, to mRNA degradation [6]. For example, as regulators of alternative splicing of apoptotic genes, RBM5, RBM6 and RBM10 are frequently deleted or mutated in lung cancer. Changes in expression of RBM5, RBM6, RBM10 differentially regulate NUMB alternative splicing to promote cell growth [25–27]. In addition, Human antigen R (HuR) competes for binding to the 3’untranslated regions (3′UTRs) of eIF4E mRNAs and regulates eIF4E at the level of mRNA stability, providing the changes in the proteome that promote the growth and survival of the malignant cells [28]. Moreover, The Musashi-2 (Msi2) RNA-binding protein is a recently identified oncogenic protein which motif binds to specific cancer-related target mRNAs, such as the tumor suppressors Pten and NUMB [29, 30], and suppresses their translation, subsequently facilitating the development and progression of numerous types of human cancer [31–33]. Therefore, the research in RBPs enriched our understanding of the molecular mechanism underlying tumorigenesis, tumor development, tumor prevention and treatment.

In this study, we discovered that RBM43 protein levels were significantly increased in ESCC compared with the matched adjacent non-tumor tissues. Furthermore, high expression levels of RBM43 were closely correlated with age and N categories, however, not with sex, tumor location, histological grade, pT status or TNM stage of patients with ESCC. Importantly, ESCC patients with high RBM43 expression exhibited significantly shorter survival times than those with low RBM43 expression. These results implied that RBM43 may play an important role in tumorigenesis and progression of ESCC. In particular, RBM43 expression can stratify patients in N0 stage and TNM stage I–II, suggesting that RBM43 may play a more pronounced role in the development and progression of ESCC during the early stage. Hence, adjuvant therapy may be beneficial to these early stage ESCC patients with high RBM43 expression. These results demonstrated that high expression of RBM43 can serve as an independent predictor of poor prognosis for ESCC patients, especially at an early stage. However, the main limitation of the study was the absence of further research to determine how RBM43 plays cancer-promoting roles in ESCC and the exact underlying mechanisms. Moreover, further study would be necessary to determine the significance of various RBM43 staining patterns among tumor and stromal cells.

The number of studies reporting the role of RBM43 in cancer is very limited and, interestingly, the existing study that is available shows contradictory results. The study found that RBM43 was significantly downregulated and its low expression was correlated with poor prognosis in hepatocellular carcinoma (HCC). Mechanistically, further experiments revealed that RBM43 was associated with the 3′UTR of Cyclin B1 mRNA and negatively regulated its stability, ultimately inhibiting the occurrence and development of HCC [34]. Compared with our study, the opposite conclusion may be due to tumor heterogeneity and the complexity of RBM43 functions. In addition, different experimental systems, including cell type and experimental methods, might also contribute to this inconsistency. Therefore, the regulatory mechanism of RBM43 in ESCC is worth further investigation.

In conclusion, the present study revealed that RBM43 is highly expressed in ESCC tissues and the increased expression of RBM43 is significantly correlated with age, N categories, and shortened survival (especially for patients with early‐stage ESCC) in ESCC. Thus, RBM43 may be a useful indicator for prognosis and adjuvant treatment for ESCC. Further studies are warranted.
